# Restrained and External-Emotional Eating Patterns in Young Overweight Children–Results of the Ulm Birth Cohort Study

**DOI:** 10.1371/journal.pone.0105303

**Published:** 2014-08-20

**Authors:** Oliver Hirsch, Viktoria J. Kluckner, Stephanie Brandt, Anja Moss, Melanie Weck, Ines Florath, Martin Wabitsch, Johannes Hebebrand, Benno G. Schimmelmann, Hanna Christiansen

**Affiliations:** 1 Philipps-University Marburg, Department of Child and Adolescent Psychology, Marburg, Germany; 2 University Hospital of Child and Adolescent Psychiatry, University of Bern, Bern, Switzerland; 3 University Medical Center Ulm, Department of Pediatrics and Adolescent Medicine, Division of Pediatric Endocrinology and Diabetes, Ulm, Germany; 4 German Cancer Research Center (DKFZ), Division of Clinical Epidemiology and Aging Research, Heidelberg, Germany; 5 University of Duisburg Essen, Department of Child and Adolescent Psychiatry and Psychotherapy, Essen, Germany; CUNY, United States of America

## Abstract

Childhood obesity is one of the greatest public health challenges in Western countries. Abnormal eating behavior is thought to be a developmental trajectory to obesity. The Eating Pattern Inventory for Children (EPI-C) has not been used for children as young as eight years, and possible associations with body weight have not yet been established. Five hundred and twenty-one children of the Ulm Birth Cohort Study (UBCS; age eight) filled out the EPI-C and BMI was assessed. Adequacy of the scales was tested with confirmatory factor analysis and a MANOVA and cluster analysis established associations between eating patterns and BMI. The factor structure of the EPI-C was confirmed (GFI = .968) and abnormal eating behavior was associated with overweight (χ^2^(8) = 79.29, p<.001). The EPI-C is a valid assessment tool in this young age group. Overweight children consciously restrain their eating.

## Introduction

Childhood obesity is one of the greatest public health challenges in Western countries [1], [2]. Its prevalence rates are as high as 20–26% [1], [3]. Childhood obesity is associated with a number of severe health risks, such as cardiovascular and endocrinological disease, type-2 diabetes, pubertas praecox (females) and tarda (males), polycystic ovarian syndrome, sleep disorders, orthopedic and gastrointestinal problems, and psychological and psychosocial impairments [1], [4]–[7].

Research has identified specific risk factors for obesity, including: crossing the 85^th^ BMI percentile in the first two years of life [5], [8]–[10]; low parental educational attainment and socioeconomic status (SES) [5], [11]; parental obesity [5], [11]–[14]; ethnic background [5], [11], [15]; sugary beverage consumption [16], [17]; and a sedentary lifestyle [11], [18]. In addition, studies attempting to identify developmental trajectories to obesity have focused on eating behaviors.

Abnormal eating behavior is thought to be a developmental pathway to obesity [19]. For instance, restrained eating may be related to cognitive self-control. When this fails, restrained eaters have a higher risk of overeating. Several studies have shown positive associations between restrained eating and overweight for adolescents (overviews in: [19]–[22]). Emotional eating refers to overeating in response to emotional stress, whereas eating in response to external food cues, as could be the case in an obesogenic environment, has been related to external eating, i. e. externally induced eating [21], [22]. Results are mixed regarding the relationship between emotional eating and weight in adolescents, with some studies finding no association, and others finding positive or negative associations [21], [22]. The relationship between external eating and weight status is unclear. Experiments suggest a positive association between food cues and intake [23], whereas studies analyzing child and adolescent eating patterns and weight status have failed to demonstrate a connection [21], [22].

To establish pathways to obesity, adequate assessment of abnormal eating patterns in children is essential. Most eating behavior questionnaires have been developed for adults, and there remains a lack of research on childhood eating patterns and their correlates [24]. A positive exception is the Eating Pattern Inventory for Children (EPI-C; [25]). The EPI-C was developed for children in fourth grade (typically ten years old), and has been used to demonstrate that children’s eating behavior is associated with their body weight (i.e., the higher the children’s relative body weight, the more reported dietary restraint) [25]. As demonstrated in research on child development, the linguistic skills that are essential for completing questionnaire self-reports increase dramatically during the elementary school years, with older children possessing more refined skills than younger ones (review: [26]). Further, Moreno, Johnson-Shelton and Boles [27] showed that school grade accounted for significant variation in BMI. Specifically, the higher the grade (from one to five) the higher the rate of overweight children, which might in turn influence ratings of eating behavior patterns. Thus, the aim of the present study is to (1) establish whether the EPI-C also adequately assesses eating behavior patterns in second graders, and (2) confirm whether the association between dietary restraint and higher body weight in these young children is consistent with that demonstrated by Schacht et al. [25] in older children.

## Methods

### Subjects & Procedure

This study is part of the eight-year follow-up study of the Ulm Birth Cohort Study (UBCS; for details of this study see [28]). The initial sample comprised 1045 healthy children (birth weight 2000 g or above), who were born at the Department of Gynecology and Obstetrics at the University of Ulm in Germany between November 2000 and November 2001. Of this initial sample, 634 participated in the eight-year follow-up carried out between March 2009 and May 2010, and of those 537 came in for further assessment to the Department. Compared to the initial sample, low parental education, smoking during pregnancy and non-German descent were less common among 8-year follow-up participants (for details see [28]).

Of those 537 children, 16 were excluded for the following reasons: incomplete data (n = 2), low IQ (<80; n = 2), or chronic somatic diseases or stimulant medication (n = 12). These exclusion criteria were set because low IQ, chronic somatic conditions, and stimulant use might influence reported eating patterns and weight. Data from 521 children were analyzed. The mean age was 8.26 (SD = 0.17). The majority of children was 8 years old (98.5%); 3 (0.6%) were 7 years and 5 (1%) were 9 years old. In addition, 48.9% were male (see Table 1 for a description of the sample).

**Table 1:** Age groups, and means of body weight, height, BMI, and BMI percentiles with standard deviations in parenthesis of participating children.

	Total sample	Boys	Girls
	N = 521	n = 255 (48.9%)	n = 266 (51.1%)
Age (years)	8.26 (0.17)	8.25 (0.16)	8.27 (0.19)
7	3 (0.6%)	2 (66.7%)	1 (33.3%)
8	513 (98.5%)	251 (48.9%)	262 (51.1%)
9	5 (1.0%)	2 (40.0%)	3 (60.0%)
Body weight (kg)	27.91 (4.94)	28.41 (5.21)	27.42 (4.62)
Body height (meters)	1.31 (0.05)	1.32 (0.05)	1.31 (0.05)
BMI	16.08 (2.04)	16.23 (2.16)	15.95 (1.92)
Under Weight	92 (17.7%)	48 (52.2%)	44 (47.8%)
Normal Weight	330 (63.3%)	151 (45.8%)	179 (54.2%)
Over Weight	99 (19%)	56 (56.6%)	43 (43.4%)

### Procedure

Letters announcing the eight-year follow-up were mailed to all parents from the original UBCS sample who participated in the six-year follow-up. Assessments were scheduled at the Department of Pediatrics and Adolescent Medicine in Ulm. Following weight and height measurements to compute BMI, children filled out the EPI-C.

Participation was voluntary and written informed consent was obtained from all parents and oral assent from children. The study was approved by the Ethics Board of the Universities Ulm and Heidelberg and by the Physicians’ Boards of the states Baden-Wuerttemberg and Bavaria.

## 

### Methods

#### Body Mass Index (BMI)

To establish under/normal weight, overweight and obesity, the standardized body mass index (BMI) was used where age is included in the formula along with height and weight [29], [30]. Body height was measured three times freestanding without shoes with a stadiometer fixed to the wall. Looking straight ahead, the Frankfurt line (virtual connection between top margin of the earhole exit to lower margin of the orbita) was horizontal during measurement. The mean score of the three assessments was calculated. Body weight was assessed with a calibrated Seca-scale. Weight was measured when children were wearing their underwear only.

BMI was calculated using the childrens’ weight and height (BMI = kg/m^2^). As recommended by the World Health Organization (WHO), underweight, normal weight, and overweight were defined according to norms for boys and girls separately, with 1 SD below the median as underweight and 1 SD above the median as overweight [31].

#### Eating Pattern Inventory for Children (EPI-C)

The EPI-C [25] investigates psychological dimensions of eating behavior as well as thoughts concerning weight in children ten years old and older. The final version of the self-report questionnaire consists of 20 items that load on four dimensions (dietary restraint, external eating, parental pressure to eat, and emotional eating), explaining a total of 62% of the variance. Items are answered on a 4-point Likert scale: 1 = *not at all*, 2 = *little*, 3 = *mostly*, and 4 = *totally*. According to the study procedure, each question was read aloud by a PhD-student or psychologist to each child separately. When a child did not answer the question after a while, the PhD-student/psychologist asked him/her if the question was clear. In case of not understanding a specific word, a synonym of the word was presented and the question could then be answered. In our experience, the questions were well comprehended, thus clarification was very rarely needed. The completion of the questionnaire takes approximately 10 minutes. Taken together, the practicability of the EPI-C in this age group was very well.

Internal consistency is satisfactory, with Cronbach’s α being 0.93 for dietary restraint, 0.80 for emotional eating, 0.74 for external eating, and 0.72 for parental pressure to eat [25]. Cronbach’s α for the current study is reported in the result section.

#### Statistical analyses

Data were analyzed using the statistical package SPSS 18.0, which incorporates AMOS 17.0, a program for structural equation modeling.

First, BMI scores and weight categories according to WHO (see above) were calculated for all children.

Second, all EPI-C items that were entered into the final factor analyses in [25] were subjected to a confirmatory factor analysis intended to replicate the established four-factor structure. We used unweighted least squares (ULS) for estimation because of non-normality in our data [32]. Several goodness of fit indices were calculated as the ULS estimation method does not calculate a global chi-square value. Nevertheless, the resulting chi square value can be set in relation to the degrees of freedom (CMIN/df). Quotients <2 suggest a good model fit and quotients between 2 and 3 signal an acceptable model fit [33]. The root mean square residual (RMR) measures the mean absolute value of the covariance residuals. Values less than 0.05 have been proposed to indicate a good model fit [32], but other authors state that a value below 0.10 signals an acceptable model fit [34], [35]. The standardized root mean square residual (SRMR) is highly recommended in the literature as an indicator of model fit. Values ≤ 0.10 signal a good model fit. The global fit index (GFI) can be considered as a measure of the proportion of variance and covariance that a given model is able to explain. The adjusted global fit index (AGFI) takes the number of parameters used in computing the GFI into account. Both indices should be >0.90, although one has to consider that they are dependent on sample size and have to be interpreted with caution [33]. Cronbach’s alpha was calculated for each subscale.

Third, one-factor univariate and multivariate general linear models (GLMs) with post hoc Tukey’s Honestly Significant Difference (HSD) tests were used to calculate differences in EPI-C subscale ratings with regard to gender and BMI subgroups.

Fourth, a one-factor univariate GLM was calculated to search for differences in body weight between the subgroups formed by cluster analysis. Huber M estimators were calculated when standard deviations were close to means [36]. Subgroups of children with different eating patterns were established by using Ward’s algorithm in hierarchical cluster analysis with squared Euclidean distances. Variables entered were the four *z*-transformed EPI-C subscale scores. The optimal number of clusters was determined by the method of Lathrop & Williams [37].

## Results

### BMI-Scores

The mean BMI score was 16.09 (SD = 2.05), and the mean BMI percentile was 44.24 (SD = 28.13). Three hundred and thirty children (63.3%, 45.8% male) had normal weight, 99 children (19%, 56.6% male) were classified as overweight, and 92 (17.7%, 52.2% male) as underweight (see Table 1 for details).

### Confirmatory factor analysis

We performed a confirmatory factor analysis to examine whether the factor structure of the EPI-C is the same in second-grade and fourth-grade children. Data of 521 subjects were entered in the analyses. The factors were allowed to correlate because this is theoretically plausible. Our model has 164 degrees of freedom. There is a preference for models with a large number of degrees of freedom. The more degrees of freedom a model has, the greater the chance that this model is rejected. If such a model is not rejected, then the obtained results can be viewed as more stable [32], [38].

The CMIN/df quotient was 1.875 and consequently signaled that the data fit well with our model. The RMR is 0.053, suggesting an acceptable model fit; the SRMR is 0.054 and also reveals a good model fit [35]. The GFI is 0.968 and can be considered to reflect a good model fit [38]. The AGFI is 0.959 and can also be considered as showing a good model fit, although one has to be cautious, as GFI and AGFI are dependent on sample size. Table 2 shows the factor loadings for the confirmatory model that are in an acceptable range (>0.40). Only the loading of item 9 on the factor “parental pressure to eat” (0.29) is lower.

**Table 2:** Standardized CFA factor loadings of the EPI-C items on their hypothesized factors.

		*Factor loadings*			
Item		1	2	3	4
	*Factor 1: Dietary restraint*				
13	It is always on my mind that I weigh too much.	0.69			
4	When I finished eating I worry about getting too fat.	0.66			
6	While eating, I am always afraid of putting on weight.	0.69			
10	I am very afraid of putting on weight.	0.65			
2	I have already tried a couple of times to eat less.	0.61			
18	I try to eat as little as possible so I don’t put on any more weight.	0.76			
14	To keep my weight, I often eat less than I would actually like to.	0.61			
19	I should try harder to lose weight.	0.74			
	*Factor 2: External eating*				
1	When I see someone eat, I also get hungry.		0.54		
20	I am often that hungry that I immediately have to eat something.		0.48		
11	I often think about food during the day.		0.68		
17	When I see food, I get hungry right away, even if it is not mealtime yet.		0.65		
8	When I am together with someone who eats a lot, I eat a lot, too.		0.53		
	*Factor 3: Parental pressure to eat*				
16	At home, I must eat whatever is put on the table.			0.98	
3	My parents always want me to eat up everything that is on my plate.			0.45	
9	At home, I am allowed to leave food I don’t like on my plate.^a^			0.29	
	*Factor 4: Emotional eating*				
5	Eating helps me when I am disappointed.				0.64
15	When I am lonely, I comfort myself with food.				0.66
7	When I am afraid or worried I eat something.				0.82
12	I eat when I am unhappy.				0.70

a Reverse scored.

The four factors in our model have low intercorrelations (<0.25) except for the correlation between external eating and emotional eating (0.56; see Table 3 for details).

**Table 3:** Factor intercorrelations.

Factor			Correlation
Dietary restraint	<–>	External eating	0.16
Dietary restraint	<–>	Parental pressure to eat	0.13
Dietary restraint	<–>	Emotional eating	0.25
External eating	<–>	Parental pressure to eat	0.24
External eating	<–>	Emotional eating	0.56
Emotional eating	<–>	Parental pressure to eat	0.14

In summary, the confirmatory factor analysis in second-grade students also supports the hypothesized structure of the EPI-C with the following Cronbach’s α coefficients: 0.87 for dietary restraint, 0.71 for external eating, 0.60 for parental pressure to eat, and 0.79 for emotional eating.

### Subscale mean differences in different BMI groups

Table 4 lists mean subscale scores of the Eating Pattern Inventory for Children (EPI-C) in underweight, normal weight, and overweight children. A one-factor multivariate GLM was calculated to compare EPI-C subscale means of eating behavior across these three groups. This resulted in an overall significant effect (Wilk’s Lambda = 0.79, F(8,1030) = 15.94, p<.001, η^2^ = 0.11), showing that the three BMI groups differ in EPI-C subscale means. Univariate analyses with Tukey’s HSD post hoc tests revealed that overweight children reported significantly more dietary restraint than underweight and normal weight children (F(2,518) = 58.18; *p*<.001; η^2^ = 0.18; Tukey’s HSD: overweight versus underweight, p<.001; overweight versus normal weight, p<.001). The effect size η^2^ can be considered high [39]. Differences on the subscale “parental pressure to eat” nearly missed statistical significance but had a negligible effect size. No substantial differences were observed on the other EPI-C subscales.

**Table 4:** Mean subscale scores of the Eating Pattern Inventory for Children (EPI-C).

			Classified by BMI percentiles^a^							
Subscales	Total		UW		NW		OW		*ANOVA* a	
	M	SD	M	SD	M	SD	M	SD	*P*	η^2^
Dietary restraint	1.59	0.67	1.37	0.46	1.48	0.57	2.17	0.79	<0.001	0.18
External eating	2.14	0.69	2.15	0.65	2.14	0.70	2.13	0.70	0.98	0.00
Parental pressure to eat	2.57	0.85	2.59	0.84	2.63	0.83	2.39	0.92	0.051	0.01
Emotional eating	1.45	0.62	1.44	0.57	1.45	0.60	1.44	0.71	0.97	0.00

*Note. M* and *SD* scores are based on the mean item response coded 1 *not at all*, 2 *little*, 3 *mostly*, 4 *totally*.

a Results of one-way analyses of variance using weight categories as independent variable; effect sizes were evaluated by means of partial η^2^.

UW = underweight, NW = normal weight, OW = overweight.

### Subscale mean differences in girls and boys

Means and standard deviations for the EPI-C, separate for girls and boys, are presented in Table 5. A one-factor multivariate GLM was calculated to compare EPI-C subscale means between girls and boys. This resulted in an overall significant effect (Wilk’s Lambda = 0.98, F(4,516) = 2.84, p = .02, η^2^ = 0.022), showing that the two groups differ in EPI-C subscale means, although the effect size (η^2^) was small. Univariate analyses revealed that boys reported a significantly higher parental pressure to eat than girls (F(1,519) = 10.21; p = .001; η^2^ = 0.019). The effect size η^2^ can also be considered small [39]. No substantial differences were observed on the other EPI-C subscales.

**Table 5:** Eating Pattern Inventory for Children (EPI-C) subscale scores by gender.

Subscales	Total (N = 521)		Boys (n = 255)		Girls (n = 266)		ANOVA^a^	
	M	SD	M	SD	M	SD	p	η^2^
Dietary restraint	1.59	0.67	1.58	0.67	1.60	0.66	.67	.00
External eating	2.14	0.69	2.12	0.68	2.16	0.70	.58	.00
Parental pressure to eat	2.58	0.85	2.70	0.88	2.46	0.81	.001	.02
Emotional eating	1.45	0.62	1.44	0.64	1.45	0.60	.77	.00

*Note. M* and *SD* scores are based on the mean item response coded 1 *not at all*, 2 *little*, 3 *mostly*, 4 *totally*.

a Results of one-way analyses of variance using weight categories as independent variable; effect sizes were evaluated by means of partial η^2^.

### Cluster analysis

We performed a cluster analysis to search for subgroups of children with different eating patterns. Compared to the original 6-cluster solution in ten-year-old children [25], a hierarchical cluster analyses in this sample suggested a simpler solution with the following five clusters: indifferent eaters (*n* = 215, 90 male), normal eaters (*n* = 112, 70 male), external eaters (*n* = 72, 31 male), restrained eaters (*n* = 57, 31 male), and emotional and external eaters (*n* = 65, 33 males).

Frequencies of children with underweight, normal weight, and overweight significantly differed across the eating behavior clusters, χ^2^(8) = 79.29; *p*<0.001. The effect size Cramér V was.39, which indicates a large effect [40]. Of the children with overweight, 57.9% belonged to the highly restrained eating cluster and 24.6% to the emotional and external eating cluster. The distribution of underweight, normal weight, and overweight status for each cluster is given in Table 6; in Table 7 means and standard deviations for the EPI-subscales according to cluster and weight type are provided.

**Table 6:** Distribution of weight status for each eating behavior subgroup.

	N	Weight status^a^			BMI-SDs			
		UW(n = 92)	NW(n = 330)	OW(n = 99)	Mean	SD	M (Huber)^b^	
1. Normal eaters	112 (21.5%)	14.3%	77.7%	8.0%	−0.33	0.74	−0.33	
2. External eaters	72 (13.8%)	26.4%	65.3%	8.3%	−0.52	0.90	−0.50	
3. Restrained eaters	57 (10.9%)	3.5%	38.6%	57.9%	0.79	1.08	0.86	
4. Emotional/externaleaters	65 (12.5%)	18.5%	56.9%	24.6%	−0.09	0.98	−0.20	
5. Indifferent eaters	215 (41.3%)	20.0%	63.7%	16.3%	−0.25	0.87	−0.28	

a According to WHO classification (de Onis et al., 2007).

b Hubeŕs M estimator.

UW = underweight, NW = normal weight, OW = overweight.

**Table 7:** Means and (standard deviations) of EPI-scales for the identified clusters and weight groups underweight (UW), normal weight (NW), overweight (OW).

		IE	NE	EE	RE	EEE
UW (n = 92)	EPI-DR	1.19 (.05)	1,25 (,08)	1.34 (.07)	2.92 (.23)	1.89 (.09)
	EPI-EE	1.88 (.07)	1.63 (.11)	2.85 (.11)	2.70 (.33)	2.55 (.13)
	EP-PP	1.99 (.09)	3.41 (.15)	3.10 (.14)	2.83 (.43)	2.80 (.17)
	EPI-EmE	1.27 (.05)	1.20 (.08)	1.31 (.08)	1.00 (.25)	2.60 (.10)
NW (n = 330)	EPI-DR	1.29 (.03)	1.30 (.04)	1.35 (.05)	2.76 (.08)	1.97 (.06)
	EPI-EE	1.98 (.04)	1.69 (.05)	2.91 (.07)	1.97 (.11)	2.89 (.08)
	EP-PP	1.96 (.05)	3.26 (.06)	3.19 (.08)	2.42 (.12)	2.96 (.09)
	EPI-EmE	1.32 (.03)	1.63 (.19)	1.42 (.23)	2.85 (.10)	2.38 (.14)
OV (n = 99)	EPI-DR	1.69 (.09)	1.63 (.19)	1.42 (.23)	2.85 (.10)	2.38 (.14)
	EPI-EE	1.78 (.08)	1.77 (.17)	2.76 (.21)	2.02 (.09)	3.06 (.13)
	EP-PP	1.63 (.11)	3.63 (.22)	3.22 (.26)	2.45 (.11)	2.89 (.16)
	EPI-EmE	1.25 (.06)	1.16 (.12)	1.12 (.15)	1.11 (.06)	2.78 (.09)

Clusters: IE = Indifferent Eaters; NE = Normal Eaters; EE = External Eaters; RE = Restraint Eaters; EEE = Emotional and External Eaters.

EPI-Scales: DR = Dietary Restraint; EE = External Eating; PP = Parental Pressure; EmE = Emotional Eating.

A one-factor univariate GLM was calculated to assess the importance of cluster membership regarding body weight, measured as standardized BMI values (Table 6). Cluster membership had a significant association with body weight (F(4, 516) = 20.76; *p*<0.001; η^2^ = 0.14). Post hoc Tukey’s HSD tests revealed that restrained eaters had a significantly higher body weight than all other subgroups (all comparisons p<.001), and that emotional and external eaters had a higher body weight than external eaters (p = .04).

## Discussion

The current study established that the EPI-C adequately assesses eating behavior patterns in eight-year-old children in the second grade. Confirmatory factor analysis showed excellent model fit and satisfactory internal consistency values for the four subscales – dietary restraint, external eating, parental pressure to eat, and emotional eating. This further confirms our initial impression that the questionnaire is feasible for eight-year-old children. Further, we were able to replicate the cluster structure for the EPI-C, though eating patterns of eight-year-olds resulted in an overall simpler structure with only five instead of six clusters. This simpler structure is not atypical for younger children, especially since the clusters emotional eaters and emotional/external eaters of the Schacht et al. [25] study were the ones that could not be separated. Thus, the EPI-C, originally designed for fourth graders [25] can also be used for younger age groups, in this case, eight-year-olds.

We further demonstrated that the association between dietary restraint and higher body weight, as established in the study by Schacht et al. [25], could be replicated in this younger sample. Children with overweight in our sample showed significantly more dietary restraint than children with underweight and normal weight; this resulted in a large effect. The high frequency of dietary restraint in children with overweight is consistent with the literature that frequently reports associations between childhood BMI and psychological factors associated with eating behavior [19], [25], [41]–[45]. Possibly, this association reflects a deliberate, but unsuccessful attempt of children with overweight to lose weight by restricting food intake. Additionally and consistent with previous research [46], [47], restrained eating itself might promote weight gain by leading to periods of increased eating. Even though this effect has not been found in professionally administered weight loss programs [48], the hypothesis of dietary restraint as a risk factor not only for disordered eating but also for overweight has not yet been rejected There is high pressure to be thin in our society, and dieting has become a normal eating pattern for many, turning dieting and weight control into salient issues well before puberty [49]. Our study shows that this eating pattern is frequent even in eight-year-olds.

Accordingly, numerous interventions have been developed to prevent childhood obesity (reviews: [7], [50]). Core elements of all interventions are healthy diet (more fruit and vegetables, reduced sugar and fat intake) and increased physical activity combined with reduced sedentary activities [1], [4], [6], [7], [50]. Our study shows that it also might be beneficial to address components such as eating behaviors, which appear to influence body weight.

### Limitations

There are several limitations to be considered. First, the participation rate in our follow-up sample is significantly lower than the initial sample. Response rates of 50% are not unusual in birth cohort studies [51]. Yet, since non-participants were more likely to have lower education, show more unhealthy behaviors (i. e. smoking during pregnancy), and are more often from non-German descent, this might have influenced results [28]. Nevertheless, the results are valid for the presented and similar study populations although the reported effect sizes of the results might not be representative for the general population. Second, only 19% of our sample were overweight, compared to up to 26% of overweight children in international studies [1], [3], which might be a consequence of the high parental education status in our study population. On the other hand, this is fairly close to international numbers, and we were able to replicate the strong association between overweight and abnormal eating patterns as shown in the Schacht et al. [25] study.

Third, this study was conducted in a single urban area, which limits its generalizability. Future studies should include children from all German-speaking countries to strengthen our current outcomes regarding the psychometric properties of the inventory.

Fourth, we were not able to determine test-retest reliability due to the lack of longitudinal data. It is of high interest whether abnormal eating behaviors as assessed using the EPI-C predict BMI in the future. If this is the case, in addition to focusing on unhealthy food choices, addressing abnormal eating behaviors should be a core element of prevention and intervention programs.

## Conclusion

The current study successfully replicated the factorial structure of the EPI-C in a large German sample of eight-year-old children. We further demonstrated a significant association between abnormal eating behaviors (restraint and emotional/external eating) and overweight in these young children. Since studies on identified risk factors demonstrate a very early onset of obesity development, early detection of abnormal eating patterns may enable health care providers to effectively address them to prevent weight gain.
